# Effect of language task demands on the neural response during lexical access: a functional magnetic resonance imaging study

**DOI:** 10.1002/brb3.133

**Published:** 2013-05-01

**Authors:** Gabriela Gan, Christian Büchel, Frédéric Isel

**Affiliations:** 1 Department of Systems Neuroscience, University Medical Center Hamburg-EppendorfHamburg, Germany; 2 Department of Psychiatry and Psychotherapy, Technische Universität DresdenDresden, Germany; 3 Institute of Psychology, Paris Descartes UniversityParis, France; 4 Laboratoire d'Excellence ‘Empirical Foundations of Linguistics’Sorbonne Paris Cité, France

**Keywords:** Associative priming, fMRI, mental lexicon, neural adaptation, semantic processing, suppression

## Abstract

This study examined the effects of linguistic task demands on the neuroanatomical localization of the neural response related to automatic semantic processing of concrete German nouns combining the associative priming paradigm with functional magnetic resonance imaging (fMRI). To clarify the functional role of the inferior frontal gyrus (IFG) for semantic processing with respect to semantic decision making compared to semantic processing per se, we used a linguistic task that involved either a binary decision process (i.e., semantic categorization; Experiment 1) or not (i.e., silently thinking about a word's meaning; Experiment 2). We observed associative priming effects indicated as neural suppression in bilateral superior temporal gyri (STG), anterior cingulate cortex (ACC), occipito-temporal brain areas, and in medial frontal brain areas independently of the linguistic task. Inferior parietal brain areas were more active for silently thinking about a word's meaning compared to semantic categorization. A conjunction analysis of linguistic task revealed that both tasks activated the same left-lateralized occipito-temporo-frontal network including the IFG. Contrasting neural associative priming effects across linguistic task demands, we found a significant interaction in the right IFG. The present fMRI data give rise to the assumption that activation of the left inferior frontal gyrus (LIFG) in the semantic domain might be important for semantic processing in general and not only for semantic decision making. These findings contrast with a recent study regarding the role of the LIFG for binary decision making in the lexical domain (Wright et al. 2011).

## Introduction

While automatic language processes are described as proceeding without awareness and producing benefits and no costs, controlled language processes are described as slower acting and requiring effort and awareness (Posner and Snyder 1975). In Psycholinguistics, behavioral evidence from priming studies on lexical access suggests that automatic lexical retrieval can be affected by controlled strategic processes depending on experimental parameters such as the stimulus onset asynchrony^1^ (SOA; De Groot 1984; Altarriba and Basnight-Brown 2007), the proportion of related prime–target pairs (PRP; De Groot 1984; Altarriba and Basnight-Brown 2007) and the linguistic task (De Groot 1983; Balota and Chumbley 1984; Balota and Lorch 1986; for reviews, Neely 1991; McNamara and Holbrook 2003). The present priming study focused on the effect of linguistic tasks on the neural response related to automatic lexical-semantic processing. In the light of a considerable controversy regarding the exact function of the left inferior frontal gyrus (LIFG) in lexical-semantic processing with respect to language-specific versus domain-general cognitive functions (decision making), we examined the functional role of the LIFG using two semantic linguistic tasks that differed in the presence of a binary decision process. Recently, Wright et al. (2011) investigated the role of the LIFG by studying the neural effects of lexical processing with respect to a binary decision process using a lexical-decision task (LDT) and a passive listening task. They showed that activation of the LIFG was larger for the LDT than the passive listening task. In contrast, passive listening elicited higher activations in a cluster composed of the right superior and middle temporal gyri (STG, MTG). At first glance, the absence of activation in the LIFG for passive listening reported by Wright et al. (2011) supports the view that LIFG may be involved in semantic decision making only. However, neural semantic priming effects (Wheatley et al. 2005; i.e., suppression of neural activation for related compared to unrelated word pairs) and neural word repetition priming effects (Chee et al. 2003) have been reported in the LIFG with linguistic tasks that did not require a binary response, namely silent reading and silently thinking about the meaning of words. The absence of consensus between the studies of Wheatley et al. (2005), Chee et al. (2003), and Wright et al. (2011) may be due to the fact that both the paradigms (Priming vs. Word presentation) and the linguistic tasks (Silently reading vs. Passive listening) did not activate semantic properties of words in the same way. In the present research, using the same experimental design and the same linguistic materials, we compared the neural response related to lexical-semantic processing by contrasting two semantic tasks that involved either a binary decision process (i.e., semantic categorization task: natural/manmade decision; Experiment 1) or not (i.e., silently thinking about a word's meaning; Experiment 2).

The role of the inferior frontal gyrus (IFG) in semantics was intensively investigated in the last two decades (for a review, Thompson-Schill et al. 1999; Bookheimer 2002; Noppeney et al. 2004). Activation of the LIFG is discussed as especially contributing to the processes required for semantic decision making (Demb et al. 1995; Gabrieli et al. 1998; Wagner et al. 2000; Roskies et al. 2001) and strategic semantic retrieval (Sylvester and Shimamura 2002).

Semantic processing using lexical tasks involving a binary decision like the LDT, semantic judgment or categorization tasks shared activations in temporal brain areas such as the inferior temporal gyrus (ITG), the MTG, and the STG, in the inferior parietal lobe (IPL), and particularly, in the LIFG (Demb et al. 1995; Roskies et al. 2001; Wagner et al. 2001; Kotz et al. 2002; Copland et al. 2003; Rossell et al. 2003; Giesbrecht et al. 2004; Raposo et al. 2006; Kuperberg et al. 2008; Ruff et al. 2008; Wright et al. 2011). Roskies et al. (2001) showed that brain activation during a two-choice semantic synonym task (i.e., subjects indicated whether two words had the same meaning) compared to a rhyme-judgment task was modulated within the LIFG. This task-driven activation of left inferior frontal regions was discussed as possibly subserving controlled “end-stage decision processes” that interact with other brain regions like the temporal cortex to access, select, gate, or retrieve semantic information stored in the lexical entries of the mental lexicon. This interpretation is in accordance with Wu et al. (2009) suggesting activation of a separate fronto-parietal network for semantic decision making and it matches the general role of frontal regions during cognitive control processes (Duncan et al. 1996; Fuster 2001; Miller and Cohen 2001; Koechlin et al. 2003). Recent neuroimaging studies showed that the neural response underlying semantic processing in semantic priming paradigms was affected by the explicit (Semantic judgment task vs. LDT) versus implicit nature of a binary linguistic decision task (Kuperberg et al. 2008; Ruff et al. 2008). Thus, semantic priming in implicit tasks was related to semantic suppression in the left anterior IFG and the right anterior orbito-frontal gyrus (Kuperberg et al. 2008), as well as in the left STG and bilateral middle frontal gyri (cf., Rissman et al. 2003). In contrast, for explicit semantic tasks, differential effects were observed with semantic suppression in the LIFG by Ruff et al. (2008), and semantic enhancement (i.e., increased neural activation for related compared to unrelated word pairs) in the left IPL by Kuperberg et al. (2008). Both studies showed consistent Task by Relatedness interactions in the left IPL with suppression for the LDT and enhancement for the semantic judgment task. Neural suppression effects for the implicit linguistic task might be explained by facilitated lexical access induced by either automatic spreading of activation that typically occur with short SOAs (i.e., 50 msec; Ruff et al. 2008), or the use of semantic expectancy strategies that occur with long SOAs (i.e., 800 msec; Kuperberg et al. 2008) as proposed before in lexical priming studies (Collins and Loftus 1975; Copland et al. 2003; Wheatley et al. 2005; Gold et al. 2006; Raposo et al. 2006). In contrast, neural enhancement effects for the explicit semantic task might be related to postlexical semantic matching mechanisms that might have been induced by the explicit nature of the task and that are especially induced by high PRPs present in both studies (cf. also, Kotz et al. 2002; Rossell et al. 2003; Raposo et al. 2006; Kuperberg et al. 2008; for reviews, Henson 2003; James and Gauthier 2006). Although the findings of Kuperberg et al. (2008) and Ruff et al. (2008) underline that linguistic task effects affect the neural response related to semantic processing, both studies cannot shed light on the function of the LIFG with respect to automatic semantic processing because semantic processing might have been affected by lexical strategies induced either by large SOAs or large PRPs. In the present study, we tested the functional role of the LIFG in automatic semantic processing with respect to a semantic decision making process controlling for SOA and PRP.

In contrast to linguistic tasks requiring a semantic or lexical decision, semantic processing using linguistic tasks that do not involve a binary decision process led primarily to activation of temporal brain regions including inferior, middle, and superior temporal regions (Petersen et al. 1988; Howard et al. 1992; Moore and Price 1999; Wright et al. 2011). The temporal brain areas are assumed to support activation of lexical entries within the mental lexicon (Howard et al. 1992; Fiebach et al. 2002). It appears that both kinds of tasks (i.e., with a binary decision or not) show neural effects in temporal brain areas but linguistic tasks involving a binary decision process seem also to involve activation of inferior frontal brain regions (cf., Wright et al. 2011). However, as pointed out before, neural semantic and repetition priming effects have been found in the LIFG using linguistic tasks requiring no binary decision (Chee et al. 2003; Wheatley et al. 2005). So, activation of the LIFG in semantic processing seems not to be restricted to complex semantic retrieval demands like in a semantic decision making task. To date, no study directly compared the neural effects of a semantic task requiring a binary decision with a semantic task that did not.

## Current Study

In the present study, we evaluated the impact of a binary semantic decision process on the neuroanatomical localization of neural associative priming effects within a fronto-parieto-temporal network (including the IFG, ITG, STG, MTG, and IPL) that is assumed to support semantic processing at word level (for a review, see Price 2000; Bookheimer 2002; Wu et al. 2009) by contrasting two semantic tasks that differed with respect to a binary semantic decision, (i.e., semantic categorization [Experiment 1], and silently thinking about a word's meaning [Experiment 2]). In both experiments, we used an associative priming paradigm with a short SOA (300 msec) and a low PRP (6.25%) to increase the chance to capture automatic lexical access of semantic representations assumed to be stored in each lexical entry. The focus lay on the functional role of the LIFG in semantic processing.

We tested whether the LIFG was specifically activated by semantic tasks involving a binary decision process. For Experiment 1, we expected associative suppression effects in temporal and frontal brain areas with a predominant activation of the LIFG shown to be especially involved during semantic decision making (Demb et al. 1995; Gabrieli et al. 1998; Wagner et al. 2000; Roskies et al. 2001; Wu et al. 2009). For Experiment 2, alternative hypotheses were formulated. If the LIFG was specifically task-related as suggested by Wright et al. (2011), then associative suppression effects should predominantly be observed in occipito-temporal regions (Petersen et al. 1988; Howard et al. 1992; Moore and Price 1999; Fiebach et al. 2002). However, if the LIFG also takes in charge lexical-semantic processing irrespective of the nature of the task, then similar results in Experiments 1 and 2 should be expected.

## Materials and Methods

### Participants

Thirty-six native speakers of German (17 females, 19 males, mean age = 26.45 ± 4.9, age range 21–41 years) recruited from a database available at the Department for Systems Neuroscience (University Medical Center Hamburg-Eppendorf, Germany) took part in the functional magnetic resonance imaging (fMRI) study. All participants were right-handed according to the Edinburgh Inventory (Oldfield 1971; mean laterality index of 97.1 ± 5.05%). All had normal or corrected-to-normal vision. None had a history of neurological or psychiatric disease. All participants gave informed consent after the experimental procedure was explained and were paid for participation. This study was approved by the research ethical committee of the University Medical Center Hamburg-Eppendorf. Eighteen of the 36 subjects (8 females and 10 males, mean age = 26.3 ± 4.6 years, age range: 21–41 years) were assigned pseudo-randomly to Experiment 1 (semantic categorization) and the remaining 18 subjects (9 females and 9 males, mean age = 26.6 ± 5.2 years, age range: 21–38 years) were assigned to Experiment 2 (silently thinking about a word's meaning). None of the subjects participating in Experiment 2 took part in Experiment 1.

### Stimuli

Critical items were 60 morphologically simplex concrete German nouns of the open class category. These items were adapted from a previous fMRI study of the neural representation of the bilingual mental lexicon (Isel et al. 2010). Half of the words (*n* = 30) referred to natural entities (e.g., Frucht^fruit^), whereas the other half (*n* = 30) referred to manmade entities (e.g., Koffer^suitcase^). The mean age of acquisition (AoA) of the critical words was 2.7 years (±0.1) for the 30 natural concrete nouns and 3.3 years (±0.1) for the 30 manmade concrete nouns. Target words were matched for word frequency (mean word frequency: natural nouns = 34 [SEM = 7.9], manmade nouns = 22 [SEM = 5.9]; CELEX database, Baayen et al. 1995), word length (mean word length: natural nouns = 5.4 letters [SEM = 0.2], manmade nouns = 5.8 letters [SEM = 0.2]) as well as for concreteness and imageability (MRC Psycholinguistics database, Coltheart 1981). Prime words in the related and unrelated conditions were matched for frequency (mean word frequency: related condition = 28 [SEM = 6.8], unrelated = 31 [SEM = 7.3]; CELEX database, Baayen et al. 1995).

In the related condition, prime–target word pairs were associatively related and therefore were matched for associative strength^2^ (mean association strength: natural nouns = 39.7% [SEM = 2.9%], manmade nouns: 42.1% [SEM = 2.3%]). In the unrelated condition, prime and target words did not present either a phonological/orthographic, morphological, or semantic/associative link. Finally, in both the related and unrelated conditions, natural noun targets were primed by natural nouns whereas manmade noun targets were primed by manmade nouns. Table 1 displays examples of word pairs in the related and unrelated conditions.

**Table 1:** Examples of word pairs in the related and unrelated conditions

Experimental condition	Prime word	Target word
Related	Saft (*juice*)	FRUCHT (*fruit*)
Unrelated	Anzeige (*announcement*)	FRUCHT (*fruit*)

English translation equivalents are shown in brackets.

In addition, we selected 420 filler pairs (300 word–word pairs, 60 “blank screen”–word pairs [12.5%; neutral condition], and 60 symbol pairs [12.5%]). Among the 300 word–word pairs, half of them were constituted of two natural nouns, whereas the other half was constituted of two manmade nouns. The nouns used for creating the filler pairs were matched on different dimensions (frequency, number of letters, imageability, and concreteness). For each word–word pair, the nature of the relation existing between the prime word and the target word was carefully inspected by two native speakers of German for ensuring that the two words did not share semantic or associative properties. All neutral pairs consisted of a blank screen of 300 msec followed by a target word (50% natural and 50% manmade words). Finally, half of the symbol pairs consisted of a series of six identical symbols (e.g., %%%%%%), whereas the other half was constituted of six different symbols consisting of the repetition of two different symbols (e.g., %$%$%$).

### Experimental design

In order to minimize the use of a possible postlexical semantic matching processing strategy, a low proportion of related prime–target pairs (PRP) was used (i.e., 6.25%). By means of a Latin square design, four experimental lists were created such that related (e.g., Saft^juice^−FRUCHT^fruit^) and unrelated (e.g., Anzeige^announcement^−FRUCHT^fruit^) pairs were balanced across four different lists. Each target was presented under both priming conditions, but no participant saw the same prime or the same target twice, thus avoiding possible practice effects that could arise from multiple presentations of an item (Slowiaczek and Pisoni 1986). Furthermore, although there was no orthographic overlap between prime and target words (i.e., a same letter at the same position in the word), primes were presented in lowercase letters, whereas targets were presented in capital letters in order to minimize sensorial match between primes and targets. In each list, the 30 related, 30 unrelated, and 420 filler pairs were organized into five sessions, with session order counterbalanced across subjects. Each session comprised 96 trials (6 related pairs, 6 unrelated pairs, and 84 filler pairs). In each session, item pairs were pseudo-randomly interspersed according to the two following constraints. First, each type of pair (related, unrelated, filler, neutral, symbol) was presented in no more than three consecutive trials. Second, no more than three pairs with natural or manmade targets were presented in succession.

### Procedure

**Figure 1:**
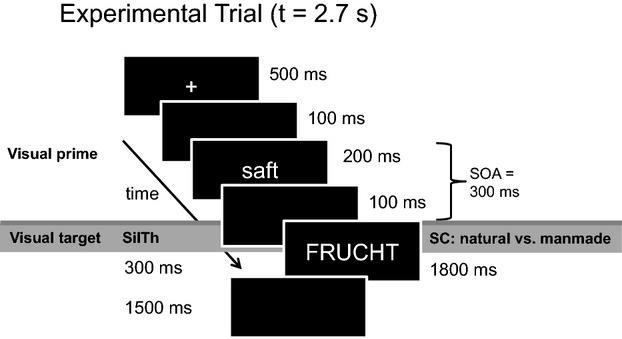
Timing (in milliseconds) used in each experimental trial of Experiment 1 (semantic categorization [SC]) and Experiment 2 (silently thinking about a word's meaning [SilTh]).

In the related, unrelated, and filler conditions, two German words were presented successively. Each word-word trial consisted of a fixation cross presented in the middle of the screen for 500 msec that was followed by (1) a blank screen presented for 100 msec, (2) a written prime word presented in lowercase letters for 200 msec, (3) a blank screen for 100 msec, and (4) a written target word presented in capital letters and remaining on the screen until the participants responded (maximal response time was limited to 1800 msec; see Fig. 1). The same timing was applied for the neutral and symbol pairs. For the neutral pairs, the prime word was replaced by a blank screen for 200 msec. For the symbol pair, the prime word was replaced by a blank screen for 200 msec, and the target was replaced by a series of either identical or different symbols. The SOA between prime and target was 300 msec. The use of a short SOA between prime and target (300 msec) ensures to reduce the risk of semantic expectancies (i.e., creation of a mental list of potential associates). The intertrial interval (ITI) separating the single trials varied between 2000 msec and 2000 msec plus one repetition time (TR; here TR = 2.37 sec) to increase the sampling rate of the blood oxygenation level-dependent (BOLD) response (Josephs et al. 1997). The stimuli were presented visually via projection to a mirror directly above the participant's head at eye level. The experimental procedure was programmed using the software presentation (Neurobehavioral Systems, http://www.neurobs.com).

Critically, Experiments 1 and 2 differed with respect to the linguistic task. However, a linguistic task involving a binary decision was used in Experiment 1 (i.e., semantic categorization), a linguistic “task” that did not require a binary decision was used in Experiment 2 (i.e., silently thinking about a word's meaning).

#### Experiment 1: semantic categorization

Participants were asked to decide whether each item presented in capital letters (i.e., the second word of each trial) was natural or manmade (i.e., semantic categorization). For the symbol pairs, participants indicated whether the series of symbols were identical or different. Participants responded using their left hand. Half of the participants (*n* = 9) used the forefinger for the response “natural” and the middle finger for the response “manmade” and the other half (*n* = 9) used the reversed pattern. The first session was preceded by a short practice session of 12 items before scanning started. Practice was repeated until participants responded without errors.

#### Experiment 2: silently thinking about a word's meaning

In the related, unrelated, neutral, and filler conditions, the trial timing was identical to the one used in Experiment 1 except for the presentation duration of the target word. The written target word was presented in capital letters for 300 msec followed by a blank screen for 1500 msec. The same timing was applied for the presentation of symbol pairs. As in Experiment 1, the prime word was replaced by a blank screen for 200 msec in the neutral and symbol trials. All other parameters (i.e., SOA, variable ITI) and the software used for stimulus presentation were equivalent to Experiment 1. In Experiment 2, inspired by Chee et al. (2003), participants were instructed to read each uppercase target-word silently and to think of its meaning (i.e., deeply process its semantic properties). Participants performed the semantic processing from the onset of the target until the next trial started. The experimental task of Experiment 2 (“silently thinking about a word's meaning”) did not require an overt behavioral decision. To ensure that participants carefully processed the critical target words, a paper–pencil postscanning recognition-test was administrated outside the scanner after the completion of the main experiment. The recognition-test was composed of 240 words. Among these words, 30 words were critical target words of the experiment (“old” target words, 1/8) whereas, the other 210 words were not (“new” target words). For each word, participants were told to indicate whether this word was presented during the experiment (“old” word) or not (“new” word). The first session was preceded by a short practice session of 12 items before scanning started. Practice was repeated once if participants did not understand the task.

Each of the five sessions lasted for ∼10 min, with 1–2 min rest between each session.

### Behavioral data analysis

#### Experiment 1

A counter module was started at the onset of the visual target presentation to register RT using presentation (Neurobehavioral Systems). We recorded both reaction times (RTs in msec) and accuracy (in %). Time-out was set at 200 msec and at 1800 msec; if the participants responded before 200 msec or after 1800 msec, the response was coded as missing. A correction procedure (mean ± 2SD) was applied on the RTs for correct responses in order to discard extreme values. RTs were then averaged in the two experimental conditions across participants and across items. Priming effects were calculated by subtracting the averaged RT in the related condition from the averaged RT in the unrelated condition by participants and by items.

#### Experiment 2

The postscanning recognition-test resulted in accuracy rates that are indicated by the percentage of hits (percentage of “old” words that were correctly recognized as “old”) and of correct rejections (percentage of “new” words that were correctly identified as “new”). We computed the mean percentage of hits and the mean percentage of correct rejections of the postscanning recognition-test per participant to gain accuracy rates.

### fMRI acquisition and analysis

All imaging data were collected with a 3.0-Tesla Magnetom TrioTim syngo MR B13 whole body system (Siemens, Erlangen, Germany). Image acquisition consisted of a fast *T*_1_-weighted sequence (localizer) and *T*_2_*-weighted sequences for functional images. Functional images were acquired in 38 axial slices using a BOLD-sensitive gradient-echo echoplanar imaging (EPI) sequence with an echo time (TE) of 30 msec, a flip angle of 90 degrees, a TR of 2.37 sec, and an acquisition bandwidth of 100 kHz. The matrix acquired was 64 × 64 with a field of view (FOV) of 192 mm^2^, resulting in an in-plane resolution of 3 mm × 3 mm. Slice thickness was 3 mm without interslice gap. Each trial had a length of 2.7 sec followed by an ITI in milliseconds varying from 2000 msec to 2000 msec + 1 TR. The functional measurements were carried out in five sessions of about 10 min length. There were 96 trials per session (480 trials, in total). In each session, about 240 volumes were recorded. For each run, the functional scanning was always preceded by five dummy scans to insure tissue steady-state magnetization. After functional scanning, a high-resolution (HR) 3D *T*_1_-weighted sequence for anatomical images was performed (12 min). HR *T*_1_ images were acquired for coregistration of the functional images (data matrix = 256 × 256, slice-thickness = 1 mm, FOV = 256 mm^2^, TR = 2.3 sec, TE = 2.98 msec). The whole experiment lasted for about 1 h. Preprocesing of fMRI data was carried out with Statistical Parametric Mapping SPM2 (Wellcome trust Centre for Neuroimaging, London, UK, http://www.fil.ion.ucl.ac.uk/spm/). First, the functional images were checked for motion-related artifacts per participant per experimental session. The exclusion criterion was set to 3 mm deviation from the initial position of the head at the beginning of a session according to the six movement parameters. Then, all functional images were corrected for slice timing, spatially realigned, normalized to the Montreal Neurological Institute (MNI) template, and smoothed using a Gaussian filter of 8 mm. A high-pass filter was used to remove low-frequency drifts.

Random-effects analyses were conducted using SPM8 (Wellcome trust Centre for Neuroimaging, London, UK, http://www.fil.ion.ucl.ac.uk/spm/). At single-subject level, we modeled each experimental condition (related, unrelated, filler pairs, neutral, and symbol trials) as separate events using the canonical hemodynamic response function (HRF) supplied by SPM8 and its temporal derivative to correct for the implied impreciseness in timing, resulting in two regressors per experimental condition. The onset of the second word of each pair (i.e., the target word, or the presentation of the symbol string) was defined as the onset of the HRF used in the regressor. For Experiment 1, we added two regressors for incorrect and missed trials to explain variance introduced by error trials. Six realignment parameters (three translation, three rotation) estimated during preprocessing were added as regressors of no interest. We computed individual contrast images for the critical conditions (related, unrelated) by subtracting the activation associated with the symbol condition from the related and unrelated condition for each linguistic task, respectively. We used the symbol condition as visual baseline condition in both tasks to subtract out any activation associated with motor responses in Experiment 1 and with activation related to basic processing of visual stimuli for both linguistic tasks. Otherwise, a comparison of both linguistic tasks would have resulted in a main effect of semantic categorization in motor brain areas.

These individual contrast estimates for the critical conditions for both linguistic tasks were subjected to a group analysis. At the group level, we ran a 2 × 2 full-factorial model with the within-subject factor Relatedness (levels: related, unrelated) and the between-subject factor Linguistic task (levels: semantic categorization, silently thinking about a word's meaning). In addition to the full-factorial model, we conducted a conjunction analysis across both linguistic tasks to examine whether both tasks recruit overlapping brain areas. For the analysis of fMRI data, the resulting statistical parameter maps were thresholded at *P* < 0.001 uncorrected. All brain areas surviving this threshold are reported in the results section. However, we restrict the discussion of data to effects found in a priori regions of interest (ROI) such as inferior and middle frontal regions, inferior parietal, middle, superior, and inferior temporal regions including the fusiform gyrus. We report the significance level at the peak level and at the cluster level corrected for multiple comparisons (family-wise error [FWE] corrected *P*-values). Only clusters of at least 25 connected voxels (i.e., 675 mm^3^) are reported. Given the a priori hypothesis of linguistic task effects in the LIFG, we also ran ROI analyses using small volume correction (SVC) implemented in SPM8. It is recommended to derive the location for the ROI from meta-analyses of functional imaging studies that explored the process of interest like “semantic processing” (Poldrack 2007; Poldrack et al. 2011). ROI analyses were performed with 15 mm spheres around the peak voxel (a) in the LIFG (MNI coordinates: *x* = −44, *y* = 24, *z* = 4, see Fig. S1 for location) showing activation for “semantic processing” in a meta-analysis provided by the Neurosynth database (source: http://neurosynth.org/terms/semantic-processing; number of implemented studies: 60), and (b) in the LIFG (MNI coordinates: *x* = −36, *y* = 33, *z* = −12) showing linguistic task effects in the Wright et al. (2011) study. Although statistical effects drawn from ROI analyses should be corrected for multiple comparisons (cf., Poldrack 2007), we used liberal significance thresholds of *P* < 0.005 (uncorrected) with at least five connected voxels to avoid Type-II errors (cf., Lieberman and Cunningham 2009).

For labeling of brain regions, we transformed MNI-coordinates to the Talairach space and used the “Talairach Daemon Client” (Lancaster et al. 1997, 2000). All coordinates were reported in MNI space in the results section.

## Results

### Experiment 1

#### Behavioral data obtained in the MRI scanner

##### Reaction times

The mean RTs averaged across participants and items and the standard errors of the mean (SEM) are displayed in Table 2. We subjected the correct RTs to an omnibus test consisting of a two-way analysis of variance (ANOVA) by participants (*F*1) and by items (*F*2) in which Relatedness (2 levels: related, unrelated) was considered as a within-subjects factor and in which List (4 levels: list 1, list 2, list 3, list 4) was considered as between-subject factor. The factor List was introduced merely to extract any variance due to the counterbalancing of critical items. A significance level of *α* = 0.05 was used for all statistical tests. The lack of any interaction with List (*Fs* < 1) indicates that the counterbalancing of items in the four experimental lists did not introduce variance in the results. Therefore, all further tests were performed on data collapsed across list. We then submitted the correct behavioral RTs to one-way ANOVAs with the within-subject factor Relatedness. The main effect of Relatedness was significant for participants (*F1*_1,17_ = 4.43, *P* = 0.5, mean square error = 1850.1), indicating that the averaged correct response times were significantly faster for the related (813 msec, SEM = 25) than for the unrelated (843 msec, SEM = 29) condition. In contrast, the main effect Relatedness was not significant for items (*F*2 < 1).

**Table 2:** Reaction times to correctly answered trials

	Subjects analysis (*F*1)	Items analysis (*F*2)
Related	813 (25)	845 (16)
Unrelated	843 (29)	864 (14)
Priming effect	30 (14)*	19 (22)n.s.

Mean reaction times (RTs; in milliseconds) for semantic categorization to target words in subjects and items analyses in each condition as well as priming effects (in milliseconds). Standard errors of the mean (SEM) are shown in brackets.

* *P* < 0.05;

n.s. refers to nonsignificant.

We included the neutral condition into the experimental design to control for inhibition effects. Behavioral analyses of RTs of the related, unrelated, and neutral condition showed that we observed facilitation but not inhibition effects. Two-tailed paired *t*-tests revealed that the mean RT of the neutral condition (894 msec [SEM: 21 msec]) was significantly longer than the mean RTs of the related (*t* = 5.337, *P* < 0.001) and the unrelated conditions (*t* = 3.082, *P* < 0.001).

##### Accuracy

The error data (in %) are presented in Table 3. Relatedness had no effect on errors **(***Fs* < 1).

**Table 3:** Task accuracy: percentages of error

	Subjects analysis (*F*1)	Items analysis (*F*2)
Related	8.7 (0.9)	8.5 (1.9)
Unrelated	8.7 (1.1)	9.1 (2.0)

Percentage of error for semantic categorization to target words in each condition in subjects and items analyses. SEM are shown in brackets.

### Experiment 2

#### Behavioral data obtained postscanning outside the MRI scanner

We assessed accuracy rates for hits (old words correctly identified as “old”) and correct rejections (new words correctly classified as “new”). The mean accuracy rates were 80% (SEM = 3%) for hits and 90% (SEM = 2%) for correct rejections. A significant positive correlation between hits and correct rejections (*r* = 0.56) was found. This correlation indicates that participants showing a high accuracy rate for hits, showed as well a high accuracy rate for correct rejections.

#### Imaging data

All results of the 2 × 2 full-factorial ANOVA and the conjunction analysis are based on whole-brain analyses surviving a significance threshold of *P* < 0.001 and represent clusters of at least 25 connected voxels. The 2 × 2 full-factorial ANOVA with the within-subject factor Relatedness and the between-subject factor Linguistic task revealed neural associative priming effects and Relatedness × Linguistic task interactions. Comparing neural activity with respect to the factor Linguistic task, no differences were apparent at a significance threshold of *P* < 0.001. The conjunction analysis revealed that semantic categorization and silently thinking about a word's meaning activated an overlapping left-lateralized network of infero-temporal and inferior frontal brain areas.

##### Neural associative priming

**Figure 2:**
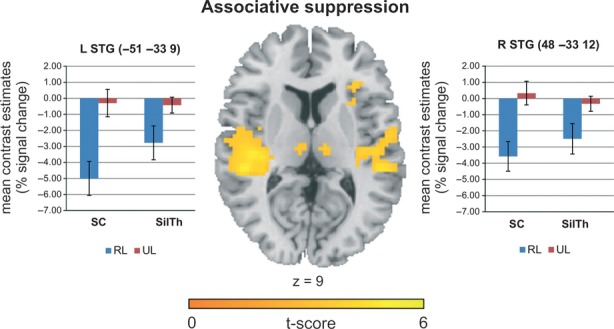
Brain areas showing neural associative suppression, that is significantly lower activation for related than for unrelated trials in native speakers of German (*n* = 36), independently of the linguistic task (*P* < 0.001 uncorrected). Mean contrast estimates (% signal change) for related (RL) compared to unrelated (UL) trials across participants are shown for the left and right superior temporal gyri (STG) for both tasks (semantic categorization [SC], silently thinking about a word's meaning [SilTh]). Error bars indicate the SEM.

In order to investigate which brain areas show neural associative priming effects independently of the factor Linguistic task, we contrasted the hemodynamic response of the unrelated with the related condition. Contrasts were assessed according to suppression of neural activity (activation of related trials < activation of unrelated trials) and to enhancement of neural activity (activation of related trials > activation of unrelated trials). We showed associative suppression effects in bilateral STG, anterior cingulate cortex (ACC), in occipito-temporal brain areas such as the lingual and the parahiccocampal gyrus and in medial frontal brain areas (BA 6/BA 9). All brain regions showing neural associative priming effects are presented in Table 4. Brain areas belonging to a priori ROIs; that is, brain regions usually involved during semantic processing as highlighted in the Introduction section (i.e., inferior and middle frontal regions, inferior parietal, middle, superior, and inferior temporal regions including the fusiform gyrus in both hemispheres) are marked in bold face. Brain areas showing neural associative suppression effects are shown in Figure 2. Additionally, we present the mean contrast estimates for related compared to unrelated trials for the neural associative priming effects in the left and right STG. No associative enhancement effects were observed. A comparison of related and unrelated trials with the neutral condition was carried out to exclude that our data were affected by inhibition effects. Consistent with the behavioral data of Experiment 1, no inhibition effects (unrelated > neutral) were observed in relevant brain areas for semantic processing (Table S1).

**Table 4:** Brain areas showing (A) neural associative suppression effects for both linguistic tasks, (B) linguistic task effects, and (C) Relatedness × Linguistic task interactions

			MNI coordinates				*P*-value (FWE-corr)	
					
Area	BA	Clustersize	*X*	*Y*	*Z*	*T*-value at peak level	Peak	Cluster
(A) Neural associative suppression (related < unrelated)								
L **Superior Temporal G**	41	482	−51	−33	9	5.22	0.022	0.000
L **Superior Temporal G**	13		−45	−21	6	5.10	0.031	
L Insula	13		−33	−27	3	4.94	0.053	
R Anterior Cingulate	32	316	6	33	27	5.19	0.024	0.000
L Anterior Cingulate	24		−6	24	24	3.95	0.636	
L Cingulate G	32		−6	18	30	3.87	0.710	
R **Superior Temporal G**	41	314	48	−33	12	5.16	0.026	0.000
R Postcentral G	40		60	−30	18	4.26	0.348	
R **Superior Temporal G**	13		45	−21	6	4.26	0.348	
L Culmen (Cerebellum)	–	323	0	−54	−3	4.68	0.116	0.000
R Parahippocampal G	19		18	−57	−6	4.48	0.202	
R Lingual G	19		30	−72	−6	4.44	0.221	
L **Medial Frontal G**	6	135	−3	−9	54	4.21	0.389	0.007
R Cingulate G	24		3	−18	42	4.13	0.454	
L **Medial Frontal G**	9	54	−21	36	30	4.15	0.437	0.141
L Lingual G	18	72	−21	−72	−12	3.96	0.625	0.067
L Declive (Cerebellum)	–		−33	−63	−12	3.84	0.742	
L Lingual G	–		−12	−72	−3	3.67	0.876	
(B) Linguistic task effect: silently thinking > semantic categorization								
L Posterior Cingulate	30	107	−9	−57	3	4.62	0.018	0.135
L Cuneus	30		−21	−75	6	4.29		0.322
L Cuneus	30		−9	−66	6	4.24		0.361
L **Inferior Parietal Lobule**	40	33	−42	−33	39	4.16	0.346	0.431
(C) Relatedness × Linguistic task interaction								
R Cingulate G	32	62	15	27	30	4.23	0.101	0.370
R Cingulate G	32		6	33	30	3.77		0.799
R **Inferior Frontal G**	45	40	45	21	6	4.22	0.256	0.382

The significance threshold was set to *P* < 0.001 with at least 25 connected voxels. The *P*-value corrected for multiple comparisons (FWE-corrected) is indicated for the peak and cluster level.

BA, Brodmann area; G, gyrus; MNI, Montreal Neurological Institute; FWE, family-wise error; L, left; R, right; a priori regions of interest are marked in bold face.

##### Linguistic task effects

No linguistic task effects could be observed in prefrontal brain areas. ROI analyses in the LIFG (a) active during semantic processing in a meta-analysis (http://www.neurosynth.org; MNI coordinates: *x* = −44, *y* = 24, *z* = 4), and (b) showing a linguistic task effect in the Wright et al. (2011) study (MNI coordinates: −36, 33, −12) did not reveal task-specific activation, even at liberal significance thresholds of *P* < 0.005 (uncorrected). Consistently, no brain region was more active for semantic categorization compared to silently thinking about a word's meaning at the specified threshold of *P* < 0.001 (uncorrected) in the full-factorial ANOVA. In contrast, higher activation was observed in occipital and inferior parietal brain areas for silently thinking compared to semantic categorization (see Table 5 section B) at *P* < 0.001 (uncorrected). Note that the individual contrast estimates for the critical conditions subjected to group-level analysis were subtracted from the symbol condition, the visual, and in the case of semantic categorization, the motor response baseline condition.

**Table 5:** Brain areas showing greater activation for the critical condition compared to the visual symbol baseline condition. (A) Brain areas showing overlapping activation for both tasks, (B) brain areas showing task activation for semantic categorization, and (C) for silently thinking about a word's meaning

			MNI coordinates				*P*-value (FWE-corr)	
					
Area	BA	Clustersize	*X*	*Y*	*Z*	*T*-value at peak level	Peak	Cluster
(A) Task conjunction: semantic categorization and silently thinking								
L **Fusiform G**	37	386	−45	−54	−15	7.18	0.000	0.000
L **Fusiform G**	20		−36	−42	−21	5.75	0.004	
L Middle Occipital G	37		−45	−69	−12	5.60	0.006	
L **Inferior Frontal G**	45	774	−51	18	15	6.63	0.000	0.000
L **Middle Frontal G**	46		−42	15	27	6.41	0.000	
L **Inferior Frontal G**	46		−48	27	15	6.25	0.001	
L Cingulate G	32	118	−6	18	48	5.64	0.005	0.012
R Pyramis (Cerebellum)	–	41	12	−84	−39	4.30	0.314	0.245
R Pyramis (Cerebellum)	–		21	−78	−45	3.79	0.779	
R Declive (Cerebellum)	–		12	−78	−30	3.68	0.870	
(B) Semantic categorization (SC)								
L **Inferior Frontal G**	46	1050	−48	30	15	9.29	0.000	0.000
L **Inferior Frontal G**	9		−45	15	24	8.76	0.000	
L **Middle Frontal G**	47		−48	36	−3	8.27	0.000	
L **Fusiform G**	37	547	−45	−54	−15	7.18	0.000	0.000
L Inferior Occipital G	18		−42	−90	−9	6.82	0.000	
L Inferior Occipital G	19		−42	−75	−12	6.66	0.000	
R Uvula (Cerebellum)	–	193	12	−87	−33	6.22	0.001	0.001
R Pyramis (Cerebellum)	–		21	−81	−45	4.81	0.079	
R Inferior Semi-Lunar Lobule (Cerebellum)	–		30	−75	−51	4.55	0.165	
L Cingulate G	32	350	−9	18	48	5.78	0.003	0.000
L Superior Frontal G	8		−3	30	51	5.42	0.011	
L Superior Frontal G	8		−6	15	57	5.00	0.044	
L **Middle Temporal G**	39	72	−39	−69	24	5.46	0.010	0.067
L **Middle Temporal G**	22	83	−60	−39	6	5.06	0.036	0.044
L **Middle Temporal G**	21		−63	−42	−6	3.43	0.975	
(C) Silently thinking about a word's meaning (silTh)								
L **Fusiform G**	37	515	−45	−51	−15	7.61	0.000	0.000
L Middle Occipital G	37		−45	−69	−9	5.96	0.002	
L **Middle Temporal G**	39		−51	−60	3	4.24	0.366	
L Superior Frontal G	6	192	−6	6	57	7.05	0.000	0.001
L Cingulate G	32		−6	18	48	5.88	0.002	
L **Inferior Frontal G**	45	1167	−51	18	15	6.63	0.000	0.000
L **Inferior Frontal G**	9		−51	15	27	6.59	0.000	
L **Middle Frontal G**	9		−39	15	27	6.50	0.000	
R Declive (Cerebellum)	–	259	39	−69	−27	5.60	0.006	0.000
R Declive (Cerebellum)	–		33	−60	−30	5.35	0.014	
R Pyramis (Cerebellum)	–		15	−81	−42	4.49	0.196	
L **Inferior Parietal Lobule**	40	228	−36	−42	39	5.37	0.013	0.000
L Precuneus	7		−27	−72	36	5.26	0.019	
L Superior Parietal Lobule	7		−30	−63	45	4.12	0.467	
L Posterior Cingulate	30	49	−9	−54	6	4.69	0.113	0.174

The significance threshold was set to *P* < 0.001 with at least 25 connected voxels. The *P*-value corrected for multiple comparisons (FWE-corrected) is indicated for the peak and cluster level.

BA, Brodmann area; G, gyrus; MNI, Montreal Neurological Institute; FWE, family-wise error; L, left; R, right; a priori regions of interest are marked in bold face.

##### Relatedness × Linguistic task interaction

**Figure 3:**
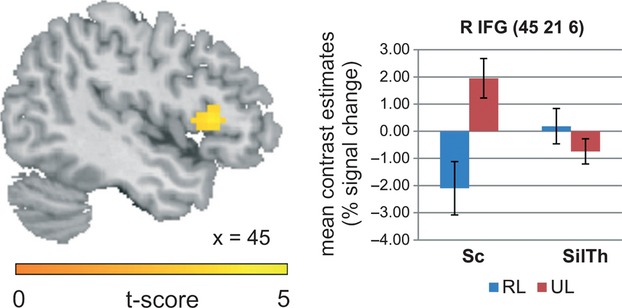
Right inferior frontal gyrus (RIFG) showing a Relatedness × Linguistic task interaction in native speakers of German (*n* = 36) at *P* < 0.001 uncorrected. Mean contrast estimates (%) for related (RL) and unrelated (UL) trials across participants for semantic categorization (SC) and silently thinking about a word's meaning (SilTh) are displayed. Error bars indicate SEM.

We evaluated the Relatedness × Linguistic task interaction by contrasting neural associative priming effects for semantic categorization with silently thinking about a word's meaning (i.e., Associative Suppression – semantic categorization > Associative Suppression – silently thinking about a word's meaning and vice versa). Relatedness × Linguistic task interactions were revealed in the right (R) IFG and the cingulate gyrus (see, Table 4 section C). This effect was significant at the specified threshold of *P* < 0.001 uncorrected, but not at a significance level corrected for multiple comparisons at peak or cluster level. The Relatedness × Linguistic task interaction in the RIFG and its mean contrast estimates are displayed in Figure 3.

##### Conjunction analysis

**Figure 4:**
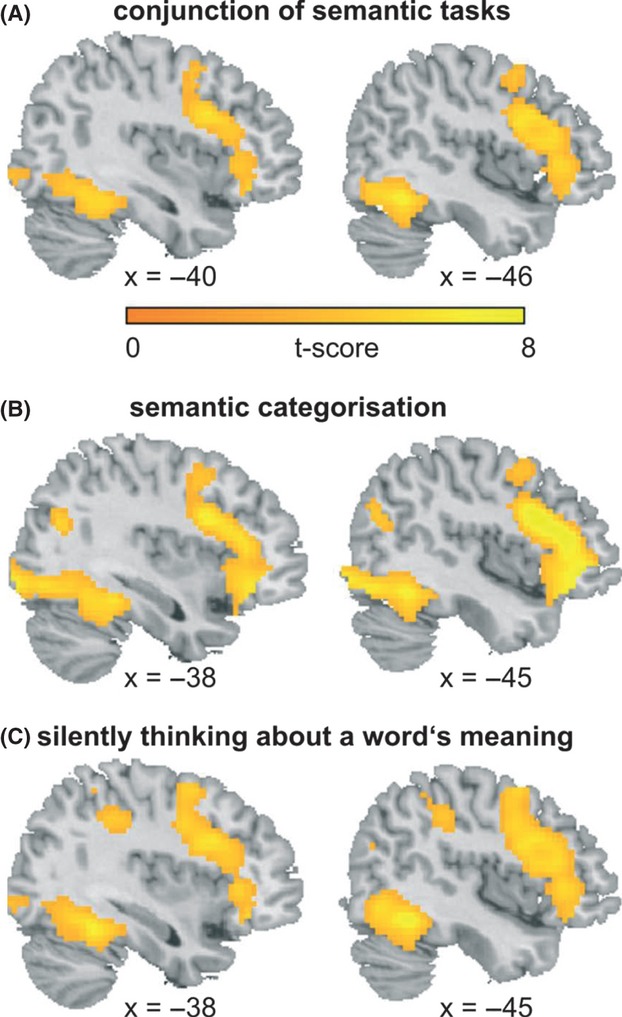
Overlapping task activation for semantic categorization and silently thinking about a word's meaning across critical conditions (related [RL], unrelated [UL]) compared to a visual baseline (A). Brain areas showing task activation for semantic categorization (B) and silently thinking about a word's meaning (C).

In addition to the 2 × 2 full-factorial ANOVA, we computed a conjunction analysis across both tasks independently of the factor Relatedness. The conjunction analysis revealed overlapping task activation in a left-lateralized network consisting of occipito-temporal brain areas including the fusiform gyrus and inferior and middle frontal brain areas (Fig. 4). All the brain areas showing overlapping activation for semantic categorization and silently thinking about a word's meaning are reported in section A of Table 5. In addition, we report the task activation ([Related + Unrelated] – Symbol) for semantic categorization and silently thinking about a word's meaning separately in sections B and C of Table 5.

## Discussion

In the present study, we investigated whether the neuroanatomical localization of neural associative priming effects within a fronto-parieto-temporal network thought to subserve semantic processing (for a review, see Price 2000; Bookheimer 2002; Wu et al. 2009) differed with respect to the presence of a binary semantic decision process. In particular, we focused on the functional role of the LIFG in semantic decision making. Linguistic task demands were systematically manipulated with respect to a binary semantic decision process in two associative priming experiments designed to elicit automatic lexical processing by controlling the SOA and PRP (cf., De Groot 1984; Balota and Lorch 1986; Neely 1991; McNamara and Holbrook 2003).

Our results provide a clear picture: the two semantic tasks activated the same left-lateralized fronto-temporal network, recruiting the fusiform gyrus, the cingulate cortex, the IFG, and MFG, irrespective of the presence of a binary decision component. No linguistic task effects could be observed in the LIFG. However, silently thinking about a word's meaning showed higher activation in inferior parietal brain areas compared to semantic categorization, but no brain area was more active for semantic categorization. Regarding associative priming effects, we found neural associative suppression effects in bilateral superior temporal brain areas, occipito-temporal, and medial frontal brain regions independently of the linguistic task. However, one brain area seemed to be selectively activated as a function of the binary decision process, namely the right IFG. At the behavioral level for semantic categorization, there was a significant 30-msec associative priming effect indicating that lexical access was facilitated (cf., Meyer and Schvaneveldt 1971). No inhibition effects were observed as expected for experimental paradigms with short SOAs and low PRPs (cf., Neely 1977). For silently thinking about a word's meaning, we observed high accuracy rates in the postscanning recognition-test with a significant positive correlation between hits and correct rejections emphasizing that participants did well process the critical words.

### Neural associative suppression effects

Observation of neural associative suppression effects in a fronto-temporal network across both tasks indicates that semantic processing was facilitated for related compared to unrelated word pairs (Copland et al. 2003; Wheatley et al. 2005; Gold et al. 2006). In the present research, the neuroanatomical activation pattern of associative suppression effects in frontal and temporal brain areas is in line with the assumption that semantic processing necessitates that prefrontal brain regions interact with temporal brain regions (cf., Roskies et al. 2001). We propose that the neural associative suppression effect in the STG and MTG likely reflects facilitated lexical access of the second word of an associatively related word pair at the level of the mental lexicon (cf., Howard et al. 1992; Fiebach et al. 2002). Temporal brain areas are discussed as being involved in accessing, selecting, gating, or retrieving semantic information stored in lexical entries of the mental lexicon (Roskies et al. 2001). Furthermore, the neural associative suppression effect observed in medial frontal brain areas (BA 6/BA 9) might reflect facilitated integration, control, and retrieval processes of semantic information that is necessary to activate semantic representations in the related compared to the unrelated condition. Activation of anterior prefrontal areas has previously been associated with integration of verbal information and control processes (e.g., Christoff and Gabrieli 2000; Prabhakaran et al. 2000), management of multiple task-relevant goals (e.g., Koechlin et al. 1999), and memory retrieval processes (Tulving et al. 1994; Schacter et al. 1996; Lepage et al. 2000; McDermott et al. 2000). Regarding neural associative suppression in the ACC, we suggest that this effect might be related to the conflict arising in the unrelated critical condition compared to no conflict in the related condition. It is well known that the ACC is activated in conflicting situations (e.g., Botvinick et al. 1999, 2001; Kerns et al. 2004). Thus, this effect is mainly related to nonlexical processes that are induced by the associative priming paradigm underlining that the paradigm worked very well.

### Linguistic task effects

Linguistic task effects were found in inferior parietal regions with higher activation for silently thinking about a word's meaning compared to semantic decision making. We suggest that this difference might be due to the fact that silently thinking about a word's meaning led to a deeper analysis of semantic content like previously observed for explicit semantic tasks (cf., Kuperberg et al. 2008; Ruff et al. 2008). No brain area was more active for semantic decision making. In contrast to Wright et al. (2011), who showed linguistic task effects with respect to binary decision making (LDT vs. Passive listening) in the LIFG, we showed overlapping activation in occipito-temporal and inferior and middle frontal regions irrespective of the binary decision. This finding suggests that the whole fronto-temporal network including the LIFG is important for activating semantic content in general irrespective of linguistic task demands. In our study, activation of the LIFG with a task that did not involve a binary decision might be explained by the fact that a “deep” semantic analysis was conducted. This could be due to the fact that we combined a paradigm favoring activation of the semantic representation of words, that is, associative priming, with a task that explicitly led the participants to deeply process the semantic properties of the words, that is, silently thinking about a word's meaning (cf., Ruff et al. 2008). Our findings are consistent with previous lexical priming studies (semantic/repetition) showing neural responses related to lexical/semantic processing in the LIFG (Chee et al. 2003; Wheatley et al. 2005) with linguistic tasks that did not involve an overt behavioral response (silently activating the meaning of words/silent reading). Activation of the LIFG irrespective of linguistic task demands converges also with a previous study of Ruff et al. (2008), who failed to show a linguistic task effect (LDT vs. Semantic judgment) in the LIFG indicating that the LIFG is active independently of the explicit or implicit nature of a linguistic task.

This is the first study that directly compared the neural response related to semantic processing in two semantic tasks, which differed with respect to semantic decision making, assessed with a linguistic paradigm tapping into automatic lexical access. Unlike in previous studies, we are convinced that the participants analyzed the semantic properties of the target words in depths in both tasks underpinned (1) by associative suppression effects in brain areas typically active during semantic processing as the STG, (2) by behavioral associative priming effects for semantic categorization, and (3) by high-accuracy rates in a postscanning recognition-test for silently thinking about a word's meaning. Altogether, our experimental choices may have contributed to be able to capture activation in the LIFG and temporal brain areas with the two linguistic tasks.

Moreover, we found a Task × Relatedness interaction in the RIFG with associative suppression for semantic categorization but not for silently thinking about a word's meaning. This interaction may be related to decision making per se, independently of activating semantic content, which would be consistent with the general role of prefrontal brain areas in decision making. However, this effect was significant at the specified significance threshold, but not after correction for multiple comparisons. Conservative significance testing in fMRI analyses has been discussed as possibly increasing the risk of committing Type-II errors compared to Type-I errors in statistical inference (Lieberman and Cunningham 2009). Thus, we suggest that the effect in the RIFG with a large cluster size of 40 voxels and a *t*-value of 4.22 is unlikely to represent a false positive. Further investigation should be conducted to disentangle the functional role of the left and right IFG in semantic processing.

## Conclusion

Left-lateralized activation of temporal and inferior frontal brain areas irrespective of linguistic task demands call into question the role of the LIFG as center of semantic decision making (cf., Demb et al. 1995; Fiez 1997; Gabrieli et al. 1998; Wagner et al. 2000; Roskies et al. 2001; Wu et al. 2009). The present fMRI data lend support to the claim that the LIFG is involved in semantic content activation in general and not especially involved during semantic decision making. In contrast, the right IFG may play a role in decision making independently of semantic processing. Further investigation would be necessary to investigate the temporal structure of the involvement of the different parts of the fronto-temporal network involved during lexical access depending on the task demands. For this purpose, combined neurophysiological and neuroimaging methods will be fruitful to precise the neurodynamics of activation within this cortical network.

## Footnotes

1 The SOA is the time interval between the onset of the first stimulus (the prime) and the second stimulus (the target) of a word pair.

2 A pretest of semantic association with 50 native speakers of German was performed in order to determine the semantic associate of each critical prime word. Participants were instructed to write as rapidly as possible the three first words that came to mind.

## Supporting Information

**Figure S1.** Location of the ROI in the LIFG derived from a meta-analysis for “semantic processing” (source: http://neurosynth.org/terms/semantic-processing). For the ROI analysis, a sphere of 15 mm was drawn around the MNI coordinates indicated in the figure.

**Table S1.** Comparison of activation for the critical conditions (related, unrelated) with the neutral condition for semantic categorization. Related, unrelated, and neutral conditions are not subtracted from the visual symbol baseline in this analysis. The significance threshold was set to *P* < 0.001 with at least 25 connected voxels. The *P*-value corrected for multiple comparisons (FWE-corrected) is indicated for the peak and cluster level.
