# Supplementary figures and images for: Effect of language task demands on the neural response during lexical access: a functional magnetic resonance imaging study
Source: Brain Behav. 2013 May 1;3(4):402–16. doi: 10.1002/brb3.133 (PMC3869681; doi:10.1002/brb3.133)

L

R

$x=-44, y=24, z=4$

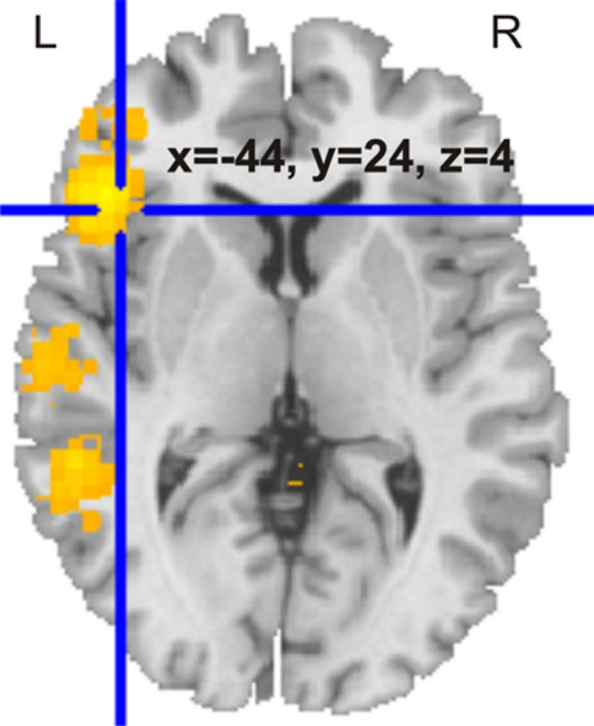

Supplement: Supplementary file 1 [file brb30003-0402-SD1.pdf]
