# Supplementary material for: Effect of language task demands on the neural response during lexical access: a functional magnetic resonance imaging study
Source: Brain Behav. 2013 May 1;3(4):402–16. doi: 10.1002/brb3.133 (PMC3869681; doi:10.1002/brb3.133)
Supplement: Supplementary file 2 [file brb30003-0402-SD2.pdf]

Supplementary materials: „Effect of language task demands on the neural response during lexical access: A functional magnetic resonance imaging study" (Gabriela Gan, Christian Büchel, and Frédéric Isel)

Table S1

Comparison of activation for the critical conditions (related, unrelated) with the neutral condition for semantic categorisation. Related, unrelated and neutral conditions are not subtracted from the visual symbol baseline in this analysis. The significance threshold was set to P <0 .001 with at least 25 connected voxels. The p-value corrected for multiple comparisons (FWE-corrected) is indicated for the peak- and cluster-level.

Area		Clustersize	MNI coordinates			T-value at peak-level	p-value at peak-level (FWE-corr)	p-value at cluster-level (FWE-corr)
			X	Y	Z			
**(A) related > neutral**								
	-							
**(B) neutral > related**								
L	Posterior cingulate G	336	-3	-54	15	6.30	0.121	0.000
R	Posterior cingulate G		12	-51	21	5.95	0.195	
R	Parahippocampal G	54	30	-30	-21	4.89	0.664	0.115
			33	-39	-6	4.40	0.897	
			24	-42	-15	3.98	0.986	
L	**Middle Temporal G**	27	-51	-6	-12	4.52	0.852	0.419
**(C) unrelated > neutral**								
L	Inferior Occipital G	558	-42	-90	-9	8.63	0.003	0.000
			-42	-78	-12	7.89	0.012	
			-45	-54	-12	6.02	0.178	
R	Inferior Occipital G	300	42	-87	-9	7.16	0.037	0.000
	Middle Occipital Gyrus		33	-96	0	5.61	0.306	
	Cuneus		24	-102	-6	5.19	0.497	
**(D) neutral > unrelated**								
	-							

We found increased activations in occipital brain areas when contrasting the unrelated with the neutral condition (UL > NT). This increased activation in brain areas usually active during processing visual stimuli seems reasonable as the visual system should be more activated in the unrelated compared to the neutral condition. In the unrelated condition, subjects saw two successively presented visual word stimuli compared to one visual1y presented word stimulus in the neutral condition. We did not find any other inhibition effects (UL > NT) in relevant brain areas for semantic processing. For neutral versus related trials, the left MTG showed increased activation for the neutral condition. This can be interpreted in

the sense of a facilitation effect (associative suppression) of the related compared to the neutral condition. As also shown in the behavioral data, no inhibition effects could be observed in the present study. Thus, the data presented in the current manuscript are not affected by inhibition but facilitation effects.

Figure S1

Figure S1: Location of the ROI in the LIFG derived from a meta-analysis for “semantic processing” (source: http://neurosynth.org/terms/semantic-processing). For the ROI analysis, a sphere of 15 mm was drawn around the MNI coordinates indicated in the figure.
